# Boat Noise Increases the Oxygen Consumption Rate of the Captive Juvenile Large Yellow Croaker, *Larimichthys crocea*

**DOI:** 10.3390/ani15050714

**Published:** 2025-03-02

**Authors:** Ruijie Xu, Shouguo Yang, Yiyu Li, Xuguang Zhang, Xianming Tang

**Affiliations:** 1 Engineering Technology Research Center of Marine Ranching, College of Oceanography and Ecological Science, Shanghai Ocean University, Shanghai 201306, China; m230501259@st.shou.edu.cn (R.X.); eyufd31@163.com (Y.L.); 2 Hainan Provincial Key Laboratory of Tropical Maricultural Technology, Hainan Academy of Ocean and Fisheries Sciences, Haikou 571126, China; yangshouguo82@163.com

**Keywords:** noise pollution, metabolic stress, optimal temperature

## Abstract

Anthropogenic noise pollution is increasingly acknowledged as a major threat to marine ecosystems, especially for sound-sensitive species, such as the large yellow croaker (*Larimichthys crocea*). While the effects of underwater noise on fish behavior and physiology have been well-documented, its influence on oxygen metabolism across varying temperatures remains poorly understood. This study examines the impact of boat noise on the oxygen consumption rate (OCR) of juvenile large yellow croakers at different temperatures, a key factor in their metabolic activity. The underwater noise generated by a fishing boat spans a broad frequency range, with a peak spectrum level of 130 dB re 1 µPa at low frequencies between 100 and 200 Hz. Our findings reveal that boat noise significantly elevates the OCR of juvenile fish, with mass-specific OCR increasing by 65.0%, 35.3%, and 28.9% at 18 °C, 25 °C, and 30 °C, respectively. Similarly, individual OCR rose by 60.7%, 35.3%, and 17.1% at these temperatures. These results demonstrate that boat noise triggers a stress response in fish, resulting in heightened metabolic demands across different seasonal conditions. Notably, the impact of boat noise on respiratory metabolism is most significant at lower temperatures. In aquatic environments with stable oxygen levels, the noise-induced rise in oxygen consumption could lead to hypoxia and provoke maladaptive behavioral changes in fish.

## Simple Summary

Coastal noise pollution threatens wild large yellow croaker (*Larimichthys crocea*) survival. Despite artificial restocking efforts, populations of this ecologically and commercially vital fish continue to decline. Our lab experiments reveal that boat noise elevates oxygen demand in juvenile croakers at high temperatures, pushing them beyond metabolic limits. These findings highlight a hidden threat: noise disrupts energy balance, particularly when fish struggle to cope with warming seas. We urge revised noise regulations in climate-vulnerable habitats to prevent croaker population collapse.

## 1. Introduction

Recently, there has been growing concern about the detrimental influence of anthropogenic underwater noise on marine life, including fish and other aquatic animals [1,2]. In fish, human-made underwater noise can mask acoustic signals, change behavior, induce physiological changes, such as high stress responses, and result in physical harm or mortality [3,4,5,6]. The extent of the impact depends on several factors, including noise levels, frequency, temporal patterns, and the distance from the source. Noise levels determine the intensity of the sound, with higher levels causing more pronounced physiological and behavioral responses in fish [7,8,9]. Frequency is also critical, as fish are most sensitive to sounds within specific frequency ranges, typically between 100 and 1000 Hz [10,11]. Temporal patterns, such as the duration, intermittency, and predictability of noise, play a significant role in determining its impact. For example, continuous noise can lead to chronic stress, while intermittent noise may cause acute stress responses [12,13,14]. Additionally, the distance from the noise source influences the sound pressure level (SPL) experienced by the fish, with closer proximity resulting in higher SPL and greater physiological impacts [7,8,9]. Temporal patterns of noise exposure, including duration, intermittency, and predictability, are critical factors influencing fish responses. For instance, prolonged exposure to continuous noise can lead to chronic stress, resulting in suppressed immune function, reduced growth, and altered behavior [12,13,14]. In contrast, intermittent noise, such as that produced by passing boats, can cause acute stress responses, including increased swimming activity and elevated cortisol levels [10,15]. Predictable noise patterns, such as regular boat traffic, may allow fish to habituate over time, whereas unpredictable noise can lead to sustained stress responses [7,8]. Recent studies have shown that temporal variability in noise exposure can significantly affect fish physiology and behavior, highlighting the need for further research on these dynamics [10,12,13,14].

The large yellow croaker, native to East Asia, is one of the most commercially important marine fish species, with extensive aquaculture development along the southeastern coast of China [16]. Nearshore floating net cages are commonly used for the rearing of large yellow croaker from juveniles to adults. At the same time, juvenile fish will also be released into the nearshore to replenish fishery stocks in the ocean. Anthropogenic underwater noise, especially originating from boat traffic, is concentrated not only in coastal waters but also extends to the open ocean. Juvenile large yellow croakers are inevitably disturbed by boat noise during their development in both artificial aquaculture cages and natural marine environments. The large yellow croaker is characterized by its large sagittae otolith, rendering it highly sensitive to sound [17]. Fish experience physiological and biochemical changes when exposed to environmental stressors, like high temperature and noise. These stress responses affect their metabolism, immunity, and behavior including oxygen demand [18,19]. As water temperature rises, fish metabolic rate increases, requiring more oxygen for critical organs, like the heart and gill [20,21]. If the temperature exceeds a certain threshold, fish may not receive enough oxygen to meet the increased demand, resulting in cellular dysfunction and tissue instability [21].

Environmental noise is a significant stressor for fish, imposing a metabolic burden. Noise pollution, especially from certain sources, like vessel noise, can increase stress hormone levels in fish. Stress-induced physiological disruptions, including immune suppression and impaired growth, are well-documented in fish under environmental stressors [22,23,24]. However, the present study focuses on oxygen consumption as a direct metabolic response to noise exposure. Similar to other organisms, fish utilize oxygen during respiration to sustain cellular respiration and provide energy for physiological processes and behaviors. Oxygen consumption rate is a crucial indicator of the fish’s metabolic activity and energy expenditure, as well as its ability to respond to environmental fluctuations [25]. Conversely, for fish, direct measurements of respiratory oxygen consumption are employed to gauge their metabolic status [26].

Temperature is a critical environmental factor that significantly influences the metabolic activity, growth, and survival of the large yellow croaker. To assess the combined effects of noise and temperature on oxygen consumption, we selected three temperature gradients (18 °C, 25 °C, and 30 °C) that reflect both natural seasonal variations and the species’ thermal tolerance range [27,28].

This study employed intermittent respiration measurement technology to investigate the effect of boat noise exposure on the oxygen consumption of juvenile yellow croaker at varying temperatures. As a coastal fish species sensitive to sound, understanding the potential impact of noise on the metabolic function of the large yellow croaker is crucial for sustainable fisheries management of this species.

## 2. Materials and Methods

### 2.1. Fish Collection and Husbandry

The juvenile large yellow croakers were all healthy and disease-free individuals and were obtained from the Zhoushan Marine Fish Hatchery (Zhejiang, China) in April 2024. After arrival at the lab of Shanghai Ocean University, to minimize the influence of housing conditions on oxygen consumption rates, fish were housed in a large indoor tank (300 × 300 × 60 cm) with a recirculating water system that maintained stable temperature (18 °C, 25 °C, 30 °C), salinity (25‰), and dissolved oxygen levels (7.97 ± 0.47 mg/L). Fish were acclimated to these conditions for at least 10 days before the experiments to reduce stress and ensure they were in a stable physiological state. A 12 h light–dark cycle was maintained to simulate natural conditions and minimize disruptions to the fish’s circadian rhythm. The fish were maintained in water bodies with sufficient space and oxygen levels, and no significant mortality occurred throughout the experiments.

### 2.2. Intermittent (Stop-Flow) Respirometry

**Figure 1:**
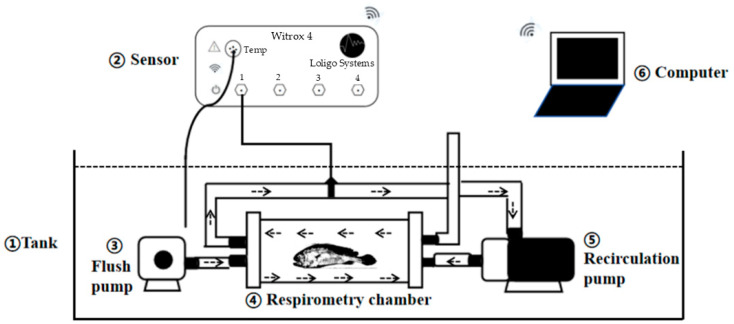
Schematic diagram of the respirometry system. ① Tank: the water reservoir that holds the experimental medium. ② Sensor (Witrox 4, Loligo^®^ Systems, Viborg, Denmark): measures the oxygen concentration and temperature in the system. It includes an integrated temperature sensor (Temp) and other monitoring components. ③ Flush pump: circulates water to ensure a steady flow through the respirometry chamber for flushing. ④ Respirometry chamber: houses the experimental organism (e.g., fish) and is the primary site for measuring oxygen consumption. ⑤ Recirculation pump: maintains water flow within the system during closed respirometry periods. ⑥ Computer: records and analyzes data collected by the sensor system, including oxygen levels and temperature. The water current flows in the direction shown by the arrows, with water being flushed through the respirometry chamber by the flush pump (③) and recirculated during measurement by the recirculation pump (⑤). The recirculation pump (⑤) was always open. During the measuring phases (30 min), the flush pump (③) was closed and opened during refresh phases (5 min), so that oxygen-saturated water from the surrounding tank was pumped into the respirometry chamber.

The impact of boat noise on the oxygen consumption rates of *L. crocea* was assessed by measuring the oxygen uptake of a group of four fish using an intermittent flow respirometer [26,29,30] equipped with a four-channel resting respirometry system. The four-channel resting respirometry system comprises four respirometry chambers (Loligo^®^ Systems, Viborg, Denmark), with each chamber measuring 20 cm in length and 10 cm in diameter, submerged parallel in a rectangular water tank filled with seawater. The rectangular tank has dimensions of 120 × 90 × 30 cm (length × width × height), with a water depth of 25 cm. During the experiment, the water temperature (18 °C, 25 °C, 30 °C) and salinity (25‰) were maintained to match the conditions of the fish captivity environment. Each respirometry chamber is circulated by two water pumps interconnected with rubber tubes (Figure 1). One pump (flush pump) operates within an external circuit to exchange of water between the respirometry chamber and surrounding water body. The second pump (recirculation pump) controls the water flow within the respirometry chamber. Intermittent respirometry allows the measurement of aquatic oxygen consumption rate through the alternating activation and deactivation of water flow via two circuits. The oxygen content within the chamber was measured using a fiber optic oxygen sensor positioned inside a recirculation pump tube.

During the experiment, oxygen levels in the chamber were recorded at a frequency of once every 5 s to ensure continuous mixing of the water within the system. Due to the potential risk of fish suffocation and mortality with extended exposure, each experiment was limited to a duration of 30 min.

### 2.3. Experiment on Oxygen Consumption

The oxygen consumption rate of juvenile fish was assessed by comparing a control group left undisturbed (no noise exposure) with a treatment group exposed to boat noise. To minimize stress during the transfer of juvenile croakers into the respirometry chambers, the fish were gently netted and allowed to acclimate in the chambers for 30 min prior to measurements. During this period, the chambers remained submerged in the main tank to maintain consistent environmental conditions, such as temperature and salinity. This acclimation period helped reduce handling stress and ensured that the fish were in a stable physiological state before oxygen consumption measurements commenced. To ensure that the observed effects on oxygen consumption rates reflected the acute impact of noise exposure, measurements were conducted immediately after sound exposure. However, the fish in the control group were undisturbed by the noise. These fish were subjected to the same experimental conditions as the treatment group (e.g., placed in a respirometry chambers, temperature gradients, and oxygen consumption measurements).

Based on the varying water temperature during the life cycle of the large yellow croaker, three water temperature gradients at 18 °C, 25 °C, and 30 °C were set to evaluate the influence of boat noise on its respiratory metabolism across different water temperature. To minimize stress on the fish, we did not simultaneously expose them to multiple temperature gradients. Instead, we took advantage of the natural increase in water temperature from April to June, raising the temperature for each experimental group. The respirometry experiments were conducted on April 29th, May 20th, and June 28th at temperatures spanning 14–23 °C, 22–27 °C, and 27–31 °C, respectively. The water temperature in the fish holding environment was maintained at 18 °C, 25 °C, and 30 °C using an aquarium heater (ARH, 500W; SUNSUN, Zhoushan, China) on the corresponding dates.

During the experiments, each temperature group consisted of 20 fish for both the control and treatment groups. For each temperature condition, we conducted two trials (one for the control group and one for the treatment group), resulting in a total of six trials across all temperature conditions. Each trial involved four fish tested simultaneously in the respirometry chambers, meaning that there were five replicates in each condition. Throughout the entire experimental process, the water quality conditions in the holding tanks for the experimental fish remained the same (as described in Section 2.2). The fish had an average total length of (10.94 ± 0.82) cm, an average standard length of (8.46 ± 0.57) cm, and an average body weight of (10.26 ± 1.97) g. No significant difference in fish body weight was observed across groups (F = 2.094, *p* = 0.128), ensuring the reliability of the experimental data.

### 2.4. Noise Exposure

The boat noise emitted by a wooden fishing boat powered by a 22-horsepower outboard engine was recorded in a mussel farming area abundant with croaker populations, including large yellow croaker. The recording was performed using a Reson hydrophone (TC4032 with an inbuilt preamplifier, sensitivity −170 dB re 1V/μPa, calibrated by the manufacturers; Teledyne Marine, Slangerup, Denmark) and an acquisition module (BK3050-A-040, 48 kHz sampling rate; Brüel & Kjær, Skodsborgvej, Denmark), approximately 20 m away from the fishing boat as it passed by, and the recording was saved as a Wav sound file. The file was played back through a portable computer with the Audacity 2.4 software. After passing through the power amplifier (Sanshui S2-350, Guangzhou, China), the signal was fed to an underwater speaker (UW30 frequency response of 0.1–10 kHz, Lubell Labs Inc., Columbus, OH, USA). An annular plate was utilized to mount the underwater speaker facing upward at the center of the bottom of a cylindrical tank with a diameter of 60 cm; the tank was filled with seawater to a depth of 70 cm.

**Figure 2:**
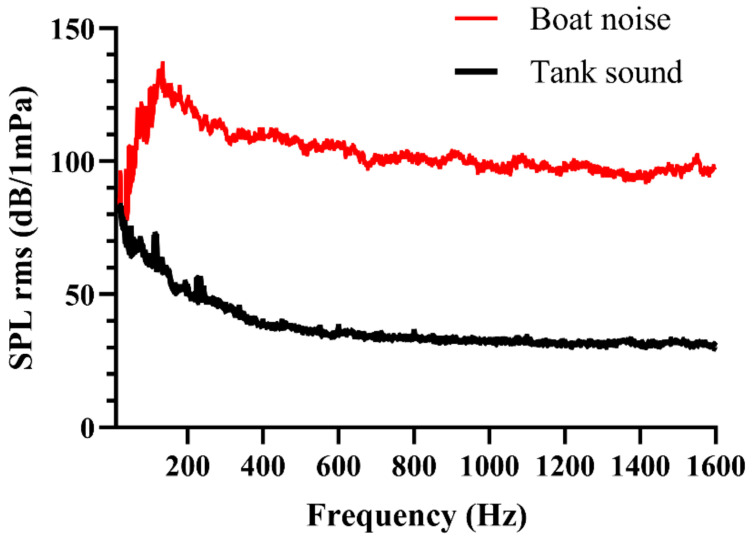
The sound pressure level of boat noise playback and ambient sound.

Figure 2 shows the mean sound pressure levels of tank boat noise obtained through spectral analysis using BK Connect software (version 2009.1) with a 1 Hz resolution and a Hanning window (66.7% overlap). Considering that most fish species are sensitive to frequencies between 100 and 1000 Hz, the analysis was limited to the 1–1600 Hz range in the spectrum, covering the auditory range of fish. The mean SPLrms from 1 to 1600 Hz was measured as (106 ± 9.1) dB re 1 μPa (mean ± S.D.), with a peak spectrum level of 130 dB re 1 µPa at low frequencies between 100 and 200 Hz at a distance of 10 cm above the speaker. The juvenile large yellow croaker was randomly exposed to boat noise for durations of 20–40 s, repeated six times within a 10 min period in the tank. This aimed to simulate the time it takes for fish to escape when encountering boat and to avoid excessive exposure that could cause harm to the fish.

### 2.5. Calculations and Analysis

The oxygen consumption rate was determined by analyzing the fluctuations in oxygen levels within the chamber during measurement intervals. The oxygen consumption rate of juvenile large yellow croaker was calculated using the following formula [31,32]:

Q = (C_0_ − C_t_) V/W/t (1)

Q_1_ = (C_0_ − C_t_) V/t (2)

where Q is the mass-specific oxygen consumption rate (mg·g^−1^·h^−1^), Q_1_ is the individual oxygen consumption rate (mg·ind^−1^·h^−1^), C_0_ and C_t_ are the dissolved oxygen content (mg/L) at the beginning and end of the experiment, respectively, V is the water volume of the respirometry chamber (L), W is the body weight of the fish (g), and t is the closed respirometry period (h).

### 2.6. Statistical Analysis

The statistical analysis of the data was performed using SPSS 26.0 software. Data were first tested for normality with the D’Agostino–Pearson omnibus normality test. Datasets that passed the normality test were analyzed with a one-way ANOVA and *t*-test, with the least significant difference (LSD) method used for the multiple comparisons test. The significance level was set at 0.05, and when differences were significant (*p* < 0.05), multiple comparisons between group means were conducted using the Duncan’s multiple comparisons test. Datasets that did not pass the normality test were analyzed with a non-parametric ANOVA on ranks (Kruskal–Wallis’s test) with a Duncan’s multiple comparisons test. Graphs and regression analysis were carried out using Origin 2024, with regression curves established (*p* < 0.05), and the curve fitting was based on maximizing the R^2^ value to obtain the optimal theoretical value. Experimental data were expressed as mean ± standard deviation (mean ± S.D.).

## 3. Results

### 3.1. Effects of Temperature on Oxygen Consumption Rate

The mass-specific oxygen consumption rate and individual oxygen consumption rate of fish exhibit a significant increase as temperature rises across three temperature levels (18 °C, 25 °C, and 30 °C; *p* < 0.05). At 18 °C, the mass-specific oxygen consumption rate was 0.20 ± 0.04 mg·g^−1^·h^−1^, and the individual oxygen consumption rate was 2.01 ± 0.46 mg·ind^−1^·h^−1^. At 25 °C and 30 °C, the mass-specific oxygen consumption rates were 0.34 ± 0.08 and 0.45 ± 0.10 mg·g^−1^·h^−1^, and the individual oxygen consumption rates were 3.57 ± 1.62 and 4.84 ± 1.55 mg·ind^−1^·h^−1^, respectively (Table 1).

### 3.2. Changes in Oxygen Consumption Rate After Boat Noise Exposure

**Figure 3:**
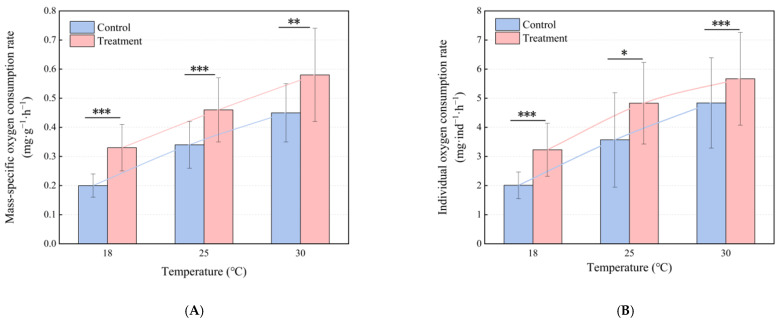
The mass-specific oxygen consumption rate (**A**) and individual oxygen consumption rate (**B**) in control fish (blue bar) and treatment fish (pink bar). The significance levels for each comparison are shown for the corresponding pairs through *t*-tests.* *p* < 0.05; ** *p* < 0.01; *** *p* < 0.001.

The presence of boat noise had a significant impact on the oxygen consumption rates of juvenile large yellow croaker. This effect was especially evident at elevated temperatures. At 18 °C, 25 °C, and 30 °C, both the mass-specific oxygen consumption rates and individual oxygen consumption rates were significantly higher in the noise treatment group compared to the control group (Table 1, Figure 3). As the temperature rose, both groups showed an increase in oxygen consumption rates, with a more pronounced rise observed in the noise-exposed group. In the control group, the mass-specific oxygen consumption rates were 0.20 ± 0.04 mg·g^−1^·h^−1^, 0.34 ± 0.08 mg·g^−1^·h^−1^, and 0.45 ± 0.10 mg·g^−1^·h^−1^ at 18 °C, 25 °C, and 30 °C, respectively. When exposed to boat noise, the mass-specific oxygen consumption rate increases by 65.0%, 35.3%, and 28.9% at the respective temperatures (Table 1, Figure 3A). In the control group, the individual oxygen consumption rates were 2.01 ± 0.46 mg·ind^−1^·h^−1^, 3.57 ± 1.62 mg·ind^−1^·h^−1^, and 4.84 ± 1.55 mg·ind^−1^·h^−1^ at 18 °C, 25 °C, and 30 °C, respectively. After exposure to boat noise, the individual oxygen consumption rates increased to 3.23 ± 0.91 mg·ind^−1^·h^−1^, 4.83 ± 1.40 mg·ind^−1^·h^−1^, and 5.67 ± 1.59 mg·ind^−1^·h^−1^, representing an increase of 60.7%, 35.3%, and 17.1%, respectively (Table 1, Figure 3B).

It is evident that as body weight increases, individual oxygen consumption rate also increases (Supplementary Table S1). In order to account for weight variations, this study focused solely on analyzing changes in mass-specific oxygen consumption rate over time.

**Figure 4:**
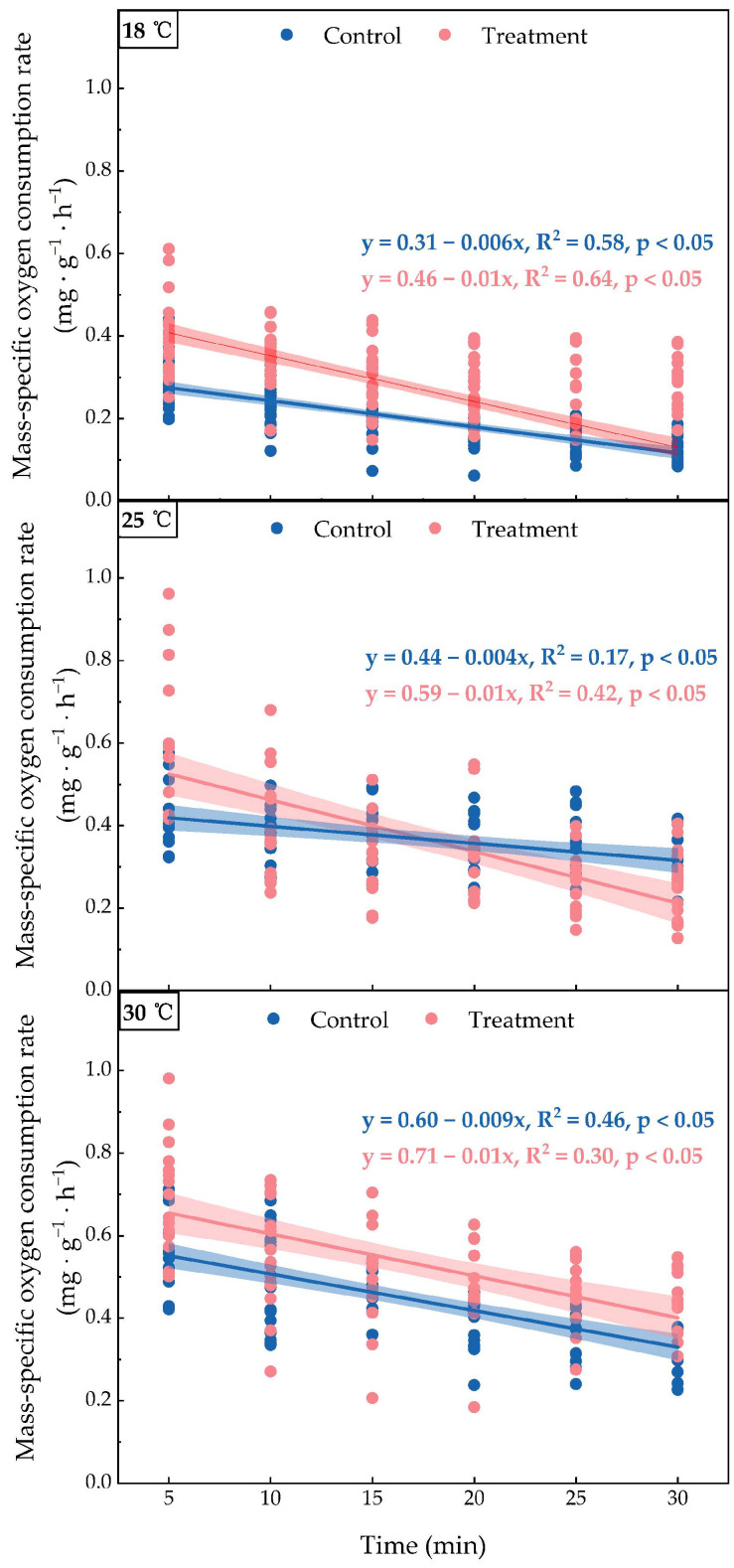
Changes in mass-specific oxygen consumption rate over time before and after exposure to boat noise. The figure illustrates the changes in mass-specific oxygen consumption rate (mg·g^−1^·h^−1^) over time (minutes) for the control and treatment groups under three temperature conditions: 18 °C, 25 °C, and 30 °C. The *y*-axis represents the mass-specific oxygen consumption rate (mg·g^−1^·h^−1^), and the *x*-axis represents time (minutes). The data are presented as box plots, with medians indicated by horizontal lines and means represented by white dots. The significance levels for each comparison are shown for the corresponding pairs through *t*-tests. * *p* < 0.05; ** *p* < 0.01; *** *p* < 0.001; ns indicates no significant difference.

At all temperature conditions, the mass-specific oxygen consumption rate of the treatment group was significantly higher than that of the control group, with a statistically significant difference (*p* < 0.05, Figure 4). However, at all temperatures, the oxygen consumption rate of both the control and treatment groups decreased over time (Figure 4). At 18 °C, the initial specific oxygen consumption rate of the treatment group was approximately 0.39 mg·g^−1^·h^−1^, while the control group had a rate of 0.29 mg·g^−1^·h^−1^ at 5 min. By 30 min, both groups had stabilized at around 0.13 mg·g^−1^·h^−1^. At 25 °C, the treatment group had an initial specific oxygen consumption rate of approximately 0.64 mg·g^−1^·h^−1^ at 5 min, higher than the control group’s rate of 0.45 mg·g^−1^·h^−1^. By 30 min, the control group’s rate had decreased to approximately 0.31 mg·g^−1^·h^−1^, while the treatment group’s rate had decreased to approximately 0.26 mg·g^−1^·h^−1^. At 30 °C, the treatment group had the highest initial specific oxygen consumption rate at 5 min, at approximately 0.72 mg·g^−1^·h^−1^, while the control group had a rate of 0.56 mg·g^−1^·h^−1^. By 30 min, the control group’s rate had decreased to approximately 0.35 mg·g^−1^·h^−1^, while the treatment group’s rate had decreased to approximately 0.43 mg·g^−1^·h^−1^.

## 4. Discussion

The oxygen metabolism of fish is predominantly controlled by internal physiological mechanisms, representing a consistent intrinsic trait that remains relatively unaffected by minor fluctuations in environmental conditions [33,34]. In similar environmental conditions, variations in measurement times, such as day or night, do not significantly affect the results (Supplementary Information). This physiological regulatory process is essential for ensuring the sustained and stable life processes of fish and for maintaining the reliability of the data collected in this experiment. While the transfer process, though its handling stress was minimized, may have influenced absolute oxygen consumption rates, the comparative validity between the noise-exposed and noise-free conditions remained unaffected.

Boat noise exposure, on the other hand, significantly elevated oxygen consumption rates in juvenile large yellow croaker, with temperature-dependent amplification (Figure 3 and Figure 4). The temperature-driven escalation in oxygen consumption was markedly amplified under noise exposure, consistent with established noise-induced metabolic demand mechanisms in fish [35].

Temperature is a crucial factor in the oxygen consumption of organisms, particularly for fish, as changes in temperature can impact metabolic activities and adaptive behavioral responses. With the ongoing increase in global temperatures, fish and other aquatic creatures must adjust to new temperature ranges in order to sustain their normal physiological functions [20,36]. Specifically, 18 °C represents the lower end of their optimal temperature range, 25 °C falls within their optimal growth range (20 °C–28 °C), and 30 °C approaches the upper limit of their thermal tolerance, mimicking conditions that may occur during warmer seasons or due to climate change [37,38]. The experimental findings show a notable increase in the oxygen consumption rate of juvenile large yellow croaker with rising temperatures, followed by a gradual decrease over time across different temperature levels. The mass-specific oxygen consumption rate increased significantly by 70.0% when the temperature rose from 18 °C to 25 °C (*p* < 0.05), compared to a 32.4% increase from 25 °C to 30 °C (*p* < 0.05). These temperatures were tested over a short period, and they reflect the range of thermal conditions that could influence fish metabolism and growth [35,39]. The highest mass-specific and individual oxygen consumption rates were seen at 30 °C (Table 1). This suggests that metabolic activity in juvenile large yellow croaker peaks at higher temperatures, approaching the upper limit of their thermal tolerance.

Exposure to noise resulted in a significant rise in oxygen consumption rates, even under extreme temperatures critical for survival. It has been demonstrated that when juvenile large yellow croakers are exposed to underwater noise, they experience a stress response that results in increased cortisol and catecholamine levels in their bodies [15,22]. Cortisol and catecholamine help the fish mobilize energy reserves by promoting the production of glucose and breaking down fats, while also inhibiting protein synthesis [15,23,24]. These metabolic changes lead to higher energy expenditure, causing an increase in the oxygen consumption rate. Initially, the elevated oxygen consumption helps the fish cope with the stress caused by noise.

When juvenile large yellow croakers were transferred to the respirometry chamber, they experienced stress responses that had not fully subsided by the time oxygen consumption measurements were taken. Over time, the fish adapt to the noisy environment by reducing unnecessary activities to conserve energy, improving aerobic metabolic processes to minimize energy loss and optimizing energy production [23,24]. They also suppress energy-intensive processes, like protein synthesis, while activating energy-generating pathways, like glycolysis and the tricarboxylic acid cycle (TCA), to generate energy [23,24]. These adaptive strategies ultimately result in a decrease in mass-specific oxygen consumption rate in juvenile large yellow croaker. As such, this resulted in a decrease in mass-specific oxygen consumption rates for both the control and treatment groups across all three temperature conditions (Figure 4). The observed metabolic downregulation—likely a compensatory mechanism to reduce oxygen demand under thermal stress—aligns with established thermoregulatory strategies in fish [40,41]. Specifically, the rate gradually decreased as time progressed, with a more significant reduction in oxygen consumption at higher temperatures. This aligns with previous research indicating that fish will make behavioral and physiological adjustments to conserve energy and prevent excessive oxygen consumption in consistent high-temperature environments [20,42].

When the environment changes, fish need to consume extra energy to sustain metabolic homeostasis and adjust to the new environmental conditions [43]. The temperature of 25 °C is generally considered optimal for the growth and metabolic activities of large yellow croaker [39,44,45]. Temperatures of 18 °C and 30 °C are below and above the optimal growth temperature of large yellow croaker (approximately 25 °C), respectively. At low temperatures, metabolic processes slow down, resulting in a relative increase in energy allocation to stress responses. In contrast, at high temperature, elevated temperatures accelerate metabolic activities, thereby increasing energy demand [43,46]. The presence of noise, as an additional environmental stressor, further exacerbates energy consumption in fish [15,47]. Consequently, the mass-specific oxygen consumption rate in the treatment group was consistently higher than that in the control group across all time points.

The mass-specific oxygen consumption rate in the noise group initially exceeded that of the control group at 25 °C but later decreased below baseline levels. This biphasic response suggests that noise exposure disrupted metabolic regulation in large yellow croaker, despite their capacity for short-term stress adaptation at optimal temperatures [22]. Our experimental design minimized pseudoreplication risks through the individual isolation of fish and randomized allocation to noise/control groups, following established protocols for aquatic bioacoustics research [48]. These findings highlight the need to evaluate chronic noise effects under climate-driven thermal shifts, as acute experimental conditions may underestimate ecological impacts.

Based on standard experimental protocols in bioacoustic research, as well as the prevalence of local fishing vessels and their representative acoustic characteristics, we used the noise generated by a 22-horsepower wooden fishing boat as the sole noise source in our study [49,50,51]. In this study, the use of a single noise source helped control noise-related confounding factors, allowing us to investigate the impact of boat noise on fish oxygen consumption. This approach is supported by relevant studies in the literature [52,53,54]. However, underwater noise in marine environments is the cumulative effect of multiple sources. Therefore, the results of this study may only be applicable to the specific noise profile used and may not fully represent the effects of other types of boat noise on fish physiology. Future studies should incorporate recordings from multiple boats with varying engine types, sizes, and operational conditions. This would provide a more comprehensive understanding of how different noise profiles affect fish metabolisms and behavior.

## 5. Conclusions

From an ecological perspective, the increased oxygen consumption rates observed in this study suggest that noise pollution could disrupt the energy balance of wild fish populations, potentially affecting their survival, reproduction, and behavior [11,55,56]. In coastal habitats, where anthropogenic noise is concentrated, these effects could exacerbate the challenges faced by already vulnerable species, such as the large yellow croaker. Conservation efforts should, therefore, prioritize the reduction of noise pollution in critical habitats, such as breeding and nursery grounds, to support the recovery and sustainability of wild fish populations.

Future research should focus on the long-term effects of noise pollution on fish physiology and behavior, particularly in aquaculture and natural environments. Additionally, studies should investigate the effectiveness of noise mitigation strategies, such as acoustic barriers or habitat restoration, in reducing the impacts of anthropogenic noise on fish populations. These efforts will provide valuable insights for the sustainable management of aquatic resources and the conservation of marine biodiversity.

## Figures and Tables

**Table 1:** The mass-specific oxygen consumption rate and individual oxygen consumption rate exposure to boat noise.

Temperature (°C)	Weight (g)		Mass-Specific Oxygen Consumption Rate (mg·g^−1^·h^−1^)		Individual Oxygen Consumption Rate (mg·ind^−1^·h^−1^)	
	Control	Noise	Control	Noise	Control	Noise
18.58 ± 0.64	10.25 ± 1.25 ^Aa^	9.99 ± 1.29 ^Aa^	0.20 ± 0.04 ^Aa^	0.33 ± 0.08 ^Ba^	2.01 ± 0.46 ^Aa^	3.23 ± 0.91 ^Ba^
25.26 ± 0.02	10.25 ± 3.16 ^Aa^	10.35 ± 1.53 ^Aa^	0.34 ± 0.08 ^Ab^	0.46 ± 0.11 ^Bb^	3.57 ± 1.62 ^Ab^	4.83 ± 1.40 ^Bb^
30.07 ± 0.09	10.75 ± 2.02 ^Aa^	10.03 ± 2.05 ^Aa^	0.45 ± 0.10 ^Ac^	0.58 ± 0.16 ^Bc^	4.84 ± 1.55 ^Ac^	5.67 ± 1.59 ^Bc^

Uppercase letter indicate significant differences (*p* < 0.05) in body weight and oxygen consumption rate of juvenile large yellow croaker between the control and noise-exposed groups under the same temperature conditions. Lowercase letters indicate significant differences (*p* < 0.05) in body weight and oxygen consumption rate of juvenile large yellow croaker across different temperatures under the same treatment conditions.

## Data Availability

The data included in this study can be provided on request from the corresponding author.

## Supplementary Materials

The following supporting information can be downloaded at: https://www.mdpi.com/article/10.3390/ani15050714/s1, Figure S1: Changes in individual oxygen consumption rate with body weight; Table S1: Two-factor ANOVA for different times of the day and different experimental dates.
