# Supplementary material for: Boat Noise Increases the Oxygen Consumption Rate of the Captive Juvenile Large Yellow Croaker, Larimichthys crocea
Source: Animals (Basel). 2025 Mar 2;15(5):714. doi: 10.3390/ani15050714 (PMC11899292; doi:10.3390/ani15050714)
Supplement: Supplementary file 1 [file animals-15-00714-s001.zip › animals-3373476-supplementary.pdf]

SUPPLEMENTARY MATERIAL

Boat noise increases the oxygen consumption rate of the captive juvenile large yellow croaker, *Larimichthys crocea*

Ruijie Xu [1,†], Shouguo Yang [2,†], Yiyu Li [1], Xuguang Zhang [1,*] and Xianming Tang [2,*]

[1] Engineering Technology Research Center of Marine Ranching, College of Oceanography and Ecological Science, Shanghai Ocean University, Shanghai 201306, China; m230501259@st.shou.edu.cn (R.X.); eyufd31@163.com (Y.L.)
[2] Hainan Provincial Key Laboratory of Tropical Maricultural Technology, Hainan Academy of Ocean and Fisheries Sciences, Haikou 571126, China; yangshouguo82@163.com
* Correspondence: zhang_xuguang@163.com (X.Z.); hn.tangxm@aliyun.com (X.T.)
† These authors contributed equally to this work and share first authorship

Oxygen Consumption Rate at Different Time Points

Experimental Design

To investigate whether variations in measurement time influence the oxygen consumption rate of juvenile fish using stop-flow respirometry, the measurements were conducted on three separate experimental dates (May 1st, May 2nd, and May 3rd) and at three times of one day (9:00 AM, 2:00 PM, and 7:00 PM). A total of 20 fish with a body weight of (59.75±17.17) g (mean ± S.D.) were measured at a water temperature of (18.58 ± 0.64)°C (mean ± S.D.).

Rusult

The results indicated that neither date nor time had a significant effect on the mass-specific oxygen consumption rate or individual oxygen consumption rate ($p>0.05$), and there was no significant interaction between date and time ($p>0.05$). A two-way ANOVA analysis was carried out to examine the impacts of different experimental dates and times on oxygen consumption rate (Table 1). Although no significant statistical difference was observed in the experimental fish (Table 1, $p>0.05$), there was still a notable positive correlation between the individual oxygen consumption rate and body weight ($R^2=0.90$, $p<0.001$), with a slope of 0.16 (Figure 1).

In Figure 1, it is evident that as body weight increases, individual oxygen consumption rate also increases. In order to account for weight variations, this study focused solely on analyzing changes in mass-specific oxygen consumption rate over time.

Table S1. Two-factor ANOVA for different times of the day and different experimental dates

	Factor	df	MS	F	P
	date	2	0.002	0.837	0.443
**Mass-specific oxygen consumption rate**	time period	1	0.004	2.080	0.160
	date*time period	1	0.001	0.306	0.585
	date	2	7.985	0.923	0.409
**Individual oxygen consumption rate**	time period	1	0.323	0.037	0.848
	date*time period	1	1.370	0.158	0.694

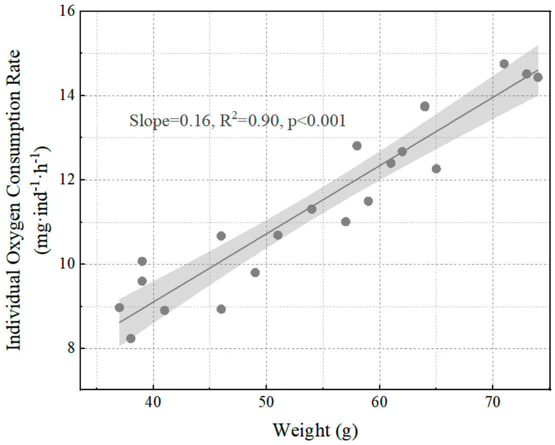

Figure S1. Changes in individual oxygen consumption rate with body weight.

Discussion

The oxygen metabolism of fish is predominantly controlled by internal physiological mechanisms, representing a consistent intrinsic trait that remains relatively unaffected by minor fluctuations in environmental conditions [1,2]. In similar environmental conditions, variations in measurement times, such as day or night, do not significantly affect the results (Table 1). This physiological regulatory process is essential for ensuring the sustained and stable life processes of fish and for maintaining the reliability of the data collected in this experiment.

References (see main paper references 33and 34)

1. Damsgaard, C.; Baliga, V.B.; Bates, E.; Burggren, W.; McKenzie, D.J.; Taylor, E.; Wright, P.A. Evolutionary and cardio-respiratory physiology of air-breathing and amphibious fishes. *Acta Physiol.* **2020**, *228*, e13406, doi:10.1111/apha.13406.
2. Reid, S.G.; Sundin, L.; Milsom, W.K. The cardiorespiratory system in tropical fishes: structure, function, and control. *Fish Physiology* **2005**, *21*, 225-275, doi:10.1016/S1546-5098(05)21006-3.
